# Characterization of Aerobic Denitrifying Bacterium *Pseudomonas mendocina* Strain GL6 and Its Potential Application in Wastewater Treatment Plant Effluent

**DOI:** 10.3390/ijerph16030364

**Published:** 2019-01-28

**Authors:** Wen Zhang, Cheng Yan, Jianing Shen, Ruping Wei, Yan Gao, Aijun Miao, Lin Xiao, Liuyan Yang

**Affiliations:** State Key Laboratory of Pollution Control and Resource Reuse, School of the Environment, Nanjing University, Nanjing 210023, China; dg1425042@smail.nju.edu.cn (W.Z.); mg1625036@smail.nju.edu.cn (C.Y.); mf1725045@smail.nju.edu.cn (J.S.); dg1725026@smail.nju.edu.cn (R.W.); gaoyan@nju.edu.cn (Y.G.); miaoaj@nju.edu.cn (A.M.); xiaolin@nju.edu.cn (L.X.)

**Keywords:** aerobic denitrification, *Pseudomonas mendocina*, nitrogen removal, response surface methodology, wastewater treatment plant effluent

## Abstract

To remove nitrate in wastewater treatment plant effluent, an aerobic denitrifier was newly isolated from the surface flow constructed wetland and identified as *Pseudomonas mendocina* strain GL6. It exhibited efficient aerobic denitrification ability, with the nitrate removal rate of 6.61 mg (N)·L^−1^·h^−1^. Sequence amplification indicated that the denitrification genes *napA*, *nirK*, *norB*, and *nosZ* were present in strain GL6. Nitrogen balance analysis revealed that approximately 74.5% of the initial nitrogen was removed as gas products. In addition, the response surface methodology experiments showed that the maximum removal of total nitrogen occurred at pH 7.76, C/N ratio of 11.2, temperature of 27.8 °C, and with shaking at 133 rpm. Furthermore, under the optimized cultivation condition, strain GL6 was added into wastewater treatment plant effluent and the removal rates of nitrate nitrogen and total nitrogen reached 95.6% and 73.6%, respectively. Thus, *P. mendocina* strain GL6 has high denitrification potential for deep improvement of effluent quality.

## 1. Introduction

Nitrate is the primary source of dissolved inorganic nitrogen in wastewater treatment plant effluent [1]. High nitrate concentration in the surface water may cause eutrophication and harmful algal blooms [2,3]. Meanwhile, excess nitrate has a serious impact on human health, such as methemoglobinemia and carcinoma [4,5]. Thus, it is of important significance to remove nitrate from contaminated water.

The main methods for nitrate removal include ion exchange, adsorption, reverse osmosis, electrodialysis, catalysis, and biological denitrification [6]. The main disadvantages of the above physicochemical approaches are high capital costs and the formation of byproducts after treatment. Moreover, these processes are expensive and unsuitable for in situ applications [7]. For these reasons, biological denitrification should be considered as an alternative process, and it is a cost-effective and environmentally friendly method of nitrate removal. It is well known that conventional biological nitrogen removal contains two stages: denitrification follows nitrification to accomplish ammonium removal. However, it is difficult to implement such a system in natural environments because nitrification needs aerobic conditions while denitrification requires anoxic conditions.

Aerobic denitrification that can simultaneously make use of nitrate and oxygen as electron acceptors was first reported in the early 1980s [8], hence it provides a potential way to get over the drawbacks of traditional biological nitrate nitrogen removal. Compared with the conventional denitrification process, aerobic denitrification is widely focused on account of its more efficient nitrogen removal and higher organic carbon degradation rate. Nevertheless, heterotrophic aerobic denitrification also has disadvantages. If the carbon source is insufficient, its nitrogen removal efficiency will decrease. To date, there have been many aerobic denitrifying bacteria isolated from the ecosystems, such as *Pseudomonas stutzeri* strain T1 [9], *Vibrio diabolicus* SF16 [10], *Pseudomonas stutzeri* YHA-13 [11], and *Enterobacter cloacae* strain HNR [12]. However, the literature describing the isolation of aerobic denitrifiers from constructed wetlands is quite limited. Moreover, previous studies on aerobic denitrifiers mainly concentrated on nitrogen removal efficiency and optimization of removal conditions [12,13,14]. However, very few studies have investigated the potential application of aerobic denitrifying bacteria for the nitrate removal in wastewater treatment plant effluent. Owing to the limitation of the conventional sewage treatment process, the effluent of municipal wastewater treatment plants still contains relatively high nitrate levels [15]. Therefore, more details should be done concerning aerobic denitrifiers applied in wastewater treatment plant effluent bioremediation.

In this study, an aerobic denitrifier, strain GL6, was isolated from surface flow constructed wetland located in a wastewater treatment plant and identified as *Pseudomonas mendocina*. The capability of aerobic denitrification by strain GL6 was evaluated, and the functional genes related to nitrogen transformation were amplified. In addition, according to response surface methodology (RSM) analysis, the factors that influence the performance of strain GL6 were comprehensively evaluated. The application of strain GL6 in wastewater treatment plant effluent was conducted to investigate its potential for use in bioaugmented treatment. The results of the current study may contribute to the development of enhanced bioremediation methods for wastewater treatment plant effluent.

## 2. Materials and Methods 

### 2.1. Isolation of Aerobic Denitrifying Bacteria

The sources of aerobic denitrifier isolation were sediment samples from the surface flow constructed wetland located in a wastewater treatment plant in Wuxi city, Jiangsu Province, China. The aerobic denitrification (AD) medium consisted of (g/L): KNO_3_, 0.720; sodium citrates, 4.08; K_2_HPO_4_, 1.00; KH_2_PO_4_, 1.00; MgSO_4_·7H_2_O, 0.200; and trace elements solution (2.00 ml/L). The final pH of the medium was adjusted to 7.20. The trace element solution contained (g/L): Na_2_EDTA, 57.1; ZnSO_4_·7H_2_O, 3.90; FeSO_4_·7H_2_O, 5.00; CaCl_2_·2H_2_O, 7.00; MnCl_2_·4H_2_O, 5.10; (NH_4_)_6_Mo_7_O_24_·4H_2_O, 1.10; CuSO_4_·5H_2_O, 1.60; and CoCl_2_·6H_2_O, 1.60 (pH = 6.00). LB (Luria–Bertani) medium included (g/L): yeast extraction, 5.00; Peptone, 10.0; and NaCl, 10.0, which was used for preculture.

Fresh sediment sample (10 mL) was inoculated into 100 mL of sterile AD medium in the 250 mL Erlenmeyer flask, and incubated at 30 °C and 160 rpm to enrich for denitrifying consortium. After three days of culture, the bacterial suspensions were transferred into fresh AD medium with 10% inoculation ratio. This process of subculture was repeated three times. The enriched bacterial suspensions were diluted in a gradient and spread on the AD medium agar plates, then cultivated at 30 °C for about 48 h. The colonies with different apparent features were picked and purified via repeated streaking on the fresh agar plates. At last, the isolates were inoculated in the AD medium to check their denitrifying activity. The strain with high nitrogen removal ability was stored in 20% glycerol solution at −80 °C.

### 2.2. Bacterial Identification and Denitrification Gene Amplification

The selected isolate GL6 with the highest TN (total nitrogen) removal efficiency was characterized and identified using standard biochemical tests in Bergey’s Manual of Determinative Bacteriology [16]. Moreover, the morphology of strain GL6 was observed by a scanning electron microscopy (FEI Quanta 250 FEG, FEI, Hillsboro, OR, USA), and the pretreatment procedure of samples was the same as the literature [17]. Universal primers 27 F and 1492R were employed to amplify the 16S rRNA of strain GL6 by PCR (polymerase chain reaction). The amplified products were purified and sequenced by Nanjing Springen Biotech Co., Ltd. (Nanjing, China). The obtained nucleotide sequence was compared with available sequences in GenBank by the BLAST (http://www.ncbi.nlm.nih.gov/blast/Blast.cgi) [18]. A phylogenetic tree was constructed in the MEGA 7.0 program (https://www.megasoftware.net/) using the neighbor-joining method with 1000 bootstrap replicates [19].

The total genomic DNA of strain GL6 was extracted using the Genomic DNA Extraction Kit (TaKaRa, Dalian, China) in accordance with the manufacturer’s instructions. The primers used for *napA*, *nirK*, *nirS*, *norB*, and *nosZ* amplification are shown in Table 1. PCR amplification was conducted in a 20 μL reaction containing 10 μL Premix Taq (TaKaRa, Dalian, China), 1.5 μL of DNA template, 0.5 μL forward primer (10 μM), 0.5 μL reverse primers (10 μM), and 7.5 μL double-distilled water. The PCR reaction conditions were as follows: 30 cycles of denaturation at 98 °C for 10 s; annealing for 30 s at 60 °C for *napA*, *norB*, at 55 °C for *nirS*, *nirK*, *nosZ*; extension at 72 °C for 60 s; the last step was extension at 72 °C for 5 min. All PCR products were analyzed with electrophoresis on a 1.5% agarose gel and sequenced as the 16S rRNA amplicon.

### 2.3. Effect of Carbon Source on Aerobic Denitrification

To seek the optimal carbon source for strain GL6, sodium acetate, sodium succinate, potassium sodium tartrate, glucose, sucrose, ethanol, and methanol were selected separately as the sole carbon source instead of sodium citrate in the AD medium, to keep a C/N ration 10. The initial NO_3_^−^-N (nitrate nitrogen) concentration was maintained at 100 mg/L. Strain GL6 was precultured overnight in LB medium. Then, 2 mL cell suspension was inoculated in a 500 mL flask containing 200 mL AD medium. The cultures were cultivated at 30 °C and 160 rpm. NO_3_^−^-N concentration and OD_600_ (optical density at 600 nm wavelength) in the cultures were determined after 48 h incubation. The above experiments were conducted in triplicate.

### 2.4. Assessment of Aerobic Denitrification Capability with Batch Experiments

Strain GL6 was precultured at 30 °C and 160 rpm using LB liquid medium for 12 h. The biomass was harvested by centrifugation (4 °C, 5 min, 6000× *g*). An inoculum concentration of 1% (*v/v*) suspension cells was aseptically transferred into the AD medium. The culture was cultivated at 30 °C and 160 rpm for 48 h. Cultures were sampled at 3 h intervals to determine OD_600_, the concentrations of NO_3_^−^-N (nitrate nitrogen), NO_2_^−^-N (nitrite nitrogen), NH_4_^+^-N (ammonium nitrogen), and TOC (total organic carbon). Each experiment was performed in triplicate.

### 2.5. Box–Behnken Design for Optimizing the Environmental Factors

The response surface methodology (RSM) is a statistical technique that establishes multiple quadratic regression equations to fit the functional relationship between factors and response values, and seeks the optimal process parameters for desirable responses through the analysis of regression equation. The TN removal rate was optimized with RSM using the Box–Behnken design. There are four independent variables with three levels: temperature (20, 30, 40 °C), pH value (5, 7, 9), C/N ratio (5, 10, 15), and shaking speed (60, 120, 180 rpm) (Table S1). A total of 29 experiments were designed with the Box–Behnken design method, and five genuine replicates were employed to estimate the experimental error. The experiments were conducted in 100 mL of AD medium in 250 mL flasks. Strain GL6 was precultured with LB liquid medium for 12 h. The biomass was harvested by centrifugation (4 °C, 5 min, 6000× *g*). An inoculum concentration of 1% (*v/v*) suspension cells was aseptically transferred into the AD medium in the flasks. After 48 h of culture, the samples were taken to determine the TN removal efficiency. Design-Expert 10.0 program was utilized to analyze the experimental results statistically.

### 2.6. Evaluation of the Nitrate Removal in Wastewater Treatment Plant Effluent by Strain GL6 under the Optimal Conditions

The wastewater used in the current experiment was the effluent of a municipal wastewater treatment plant (A2/O process) in Wuxi, China. The wastewater contained (mg/L): NO_3_^−^-N, 11.94 ± 0.63; NH_4_^+^-N, 0.13 ± 0.06; COD (chemical oxygen demand), 15.26 ± 2.3; TN, 12.78 ± 0.85. Strain GL6 was cultured in LB medium for 12 h, then it was inoculated into the 250 mL flask containing 100 mL filter sterilized wastewater with 10% inoculation ratio. Culture was supplemented with sodium citrate (at optimized concentration) as carbon source and cultivated under the optimal conditions above. Concentrations of NO_3_^−^-N, NH_4_^+^-N, and TN were determined periodically. Raw wastewater without the addition of carbon source and bacterial inoculum served as control. All experiments were performed in triplicate.

### 2.7. Analytical Methods

The growth of GL6 was measured using a spectrophotometer at a wavelength of 600 nm (UV-1800 Shimadzu, Kyoto, Japan). The concentrations of NO_3_^−^-N, NH_4_^+^-N, NO_2_^−^-N, and TN were detected by the standard methods [25]. TOC was determined with a TOC analyzer (Shimadzu, Kyoto, Japan). Intracellular nitrogen content was calculated by subtracting the TN of the inoculated medium following centrifugation (4 °C, 5 min, 6000× *g*) from the TN of noncentrifuged medium.

## 3. Results and Discussion

### 3.1. Isolation and Identification of Strain GL6

**Figure 1:**
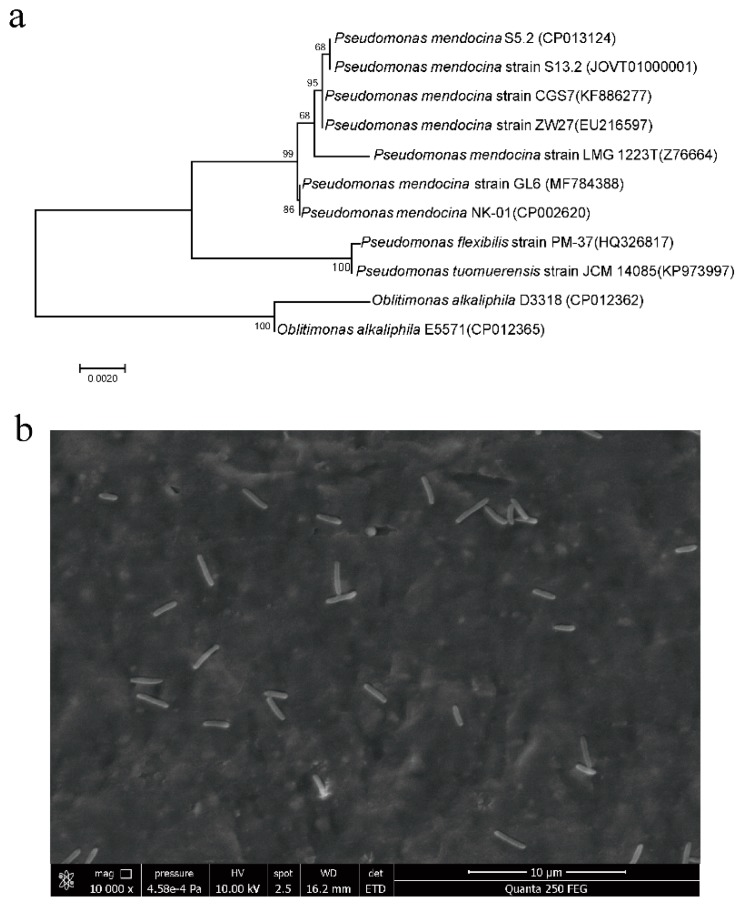
(**a**) The phylogenetic tree derived from neighbor-joining analysis of partial 16S rRNA sequences of *Pseudomonas mendocina* strain GL6. (**b**) Scanning electron microscopy picture of the strain GL6.

Strain GL6 was chosen from 50 purified isolates due to the highest TN removal efficiency. Almost the entire 16S rRNA sequence (1502 bp) of strain GL6 was obtained and submitted to the GenBank under the accession number MF784388. The BLAST results indicated that strain GL6 was closely related to *P. mendocina* NK-01 with a similarity of 99%. A neighbor-joining phylogenetic tree was constructed based on 16S rRNA of strain GL6 and some other phylogenetically related strains (Figure 1a). Results revealed that the strain GL6 and *P. mendocina* were in the same group. The strain is gram-negative and rod-shaped (approximately 0.4–0.5 × 1.5–2.0 μm) (Figure 1b). The colony was rounded and whitish in agar plate. Therefore, the screened strain GL6 was identified as *P. mendocina* based on phenotypic and genotypic characteristics.

### 3.2. PCR Amplification of Denitrification Genes

**Figure 2:**
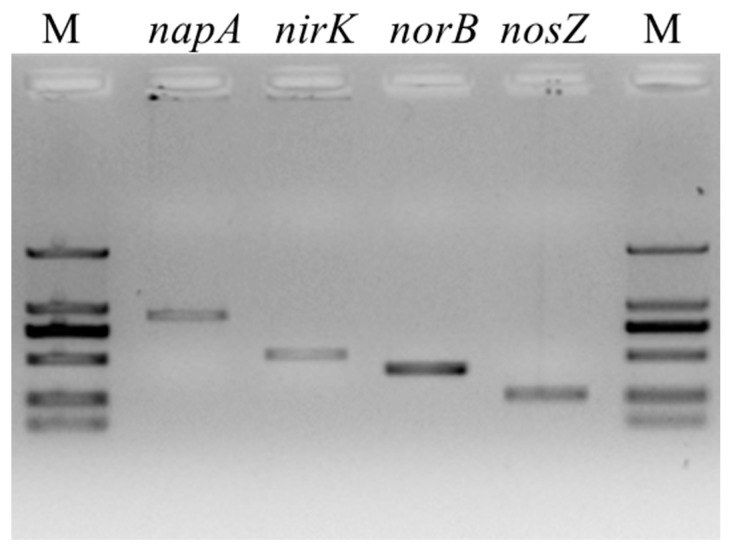
Amplification results of *napA*, *nirK*, *norB*, and *nosZ* genes from *P. mendocina* strain GL6. (M: DL 2000 DNA marker).

As shown in Figure 2, an 876 bp length fragment of *napA* gene was amplified successfully from strain GL6, which proved the ability for nitrate reduction under aerobic conditions. The BLAST results indicated that the *napA* gene sequence similarity reached 99% between strain GL6 and *Pseudomonas mendocina* NK-01 (CP002620.1). This result further confirmed that the amplified gene fragment was *napA*. The major biological denitrification pathway was reduction of NO_3_^−^-N to N_2_ in the chemical steps of NO_3_^−^-N→NO_2_^−^-N→NO→N_2_O→N_2_, catalyzed by nitrate reduction (NR), nitrite reductase (NIR), nitric oxide reductase (NOR), and nitrous oxide reductase (NOS), respectively [26]. The *napA* gene encodes the large catalytic subunit of NAP (periplasmic nitrate reductase). NAP can function under aerobic or anaerobic conditions but is primarily expressed under aerobic conditions. NAP is not dependent on nitrate transport through the cytoplasmic membrane and can catalyze the reduction of nitrate to nitrite in aerobic denitrification [27]. It is reported that many aerobic denitrifying bacteria such as *Thiosphaera pantotropha* [28], *Pseudomonas stutzeri* strain T1 [9], *Klebsiella pneumoniae* CF-S9 [29], and *Pseudomonas aeruginosa* PCN-2 [30] express the *napA* gene. Hence, *napA* is usually employed as a functional marker to identify aerobic denitrifying bacteria [31].

Nitrite reductase that catalyzes the reduction of nitrite to NO is the key enzyme in the denitrification process. There are two types of nitrite reductase: a copper and a cytochrome cd_1_-containing nitrite reductase encoded by the genes *nirK* and *nirS*, respectively [32]. In the present experiment, a 514 bp length fragment of *nirK* was successfully amplified while the amplification of *nirS* failed. The BLAST results also showed that the *nirK* gene sequence of strain GL6 was closely related to that of *Pseudomonas mendocina* S5.2 (CP013124.1) (similarity 99%). The results suggest that strain GL6 could only express copper nitrite reductase. This was in agreement with the claim that the two nitrite reductases could not be expressed simultaneously in the same bacteria. [33]. In addition, a 389 bp length fragment of the *norB* gene and a 250 bp length fragment of the *nosZ* gene were amplified from strain GL6 (Figure 2). 

The *norB* and *nosZ* genes encode the NOR and NOS enzymes, respectively [26]. NOR catalyzes the reduction of nitric oxide to nitrous oxide at the outside of the cytoplasmic membrane. The BLAST results indicated that the *norB* gene sequence of strain GL6 was closely related to that of *Pseudomonas mendocina* NK-01 (CP002620.1) (similarity 99%). The last denitrification step is the reduction of nitrous oxide to nitrogen, which is catalyzed by NOS. The presence of the *nosZ* gene in strain GL6 may reduce the accumulation of greenhouse gas N_2_O. The BLAST results indicated that *nosZ* from strain GL6 and *Pseudomonas mendocina* strain NCTC 10897 (LR134290.1) may have a relatively close phylogenetic relationship.

The successful amplification of the *napA, nirK, norB, nosZ* genes provided additional proof of aerobic denitrification by strain GL6, and further demonstrated that the nitrate removal of strain GL6 could be accomplished by the complete aerobic denitrification pathway.

### 3.3. Influence of Carbon Source on Denitrification

**Figure 3:**
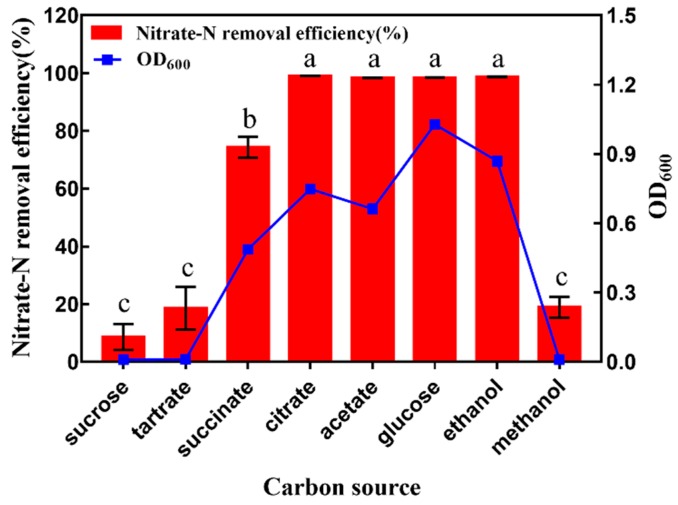
Effects of carbon source on cell growth and nitrate removal efficiency by *Pseudomonas mendocina* strain GL6 after 48 h incubation. Values are mean ± SD (standard deviation) for three replicates. Same letters indicate no significant difference between treatments for different carbon sources according to LSD (least significant difference) test (*p* ≤ 0.05). OD_600_: optical density at 600 nm wavelength.

Carbon source is one of the important factors influencing cell growth and aerobic denitrification ability, for it serves as energy and electron donor source [34]. Thus, different carbon sources affected the bacterial growth and nitrate-N removal efficiency significantly (Figure 3). When citrate, acetate, glucose, and ethanol were used as the sole carbon source, strain GL6 grew well and nitrate-N removal efficiency was almost 100%. However, with sucrose, tartrate, and methanol as the sole carbon source, the growth of strain GL6 was extremely small, and the nitrate-N removal efficiency was less than 20%. Methanol is a single carbon source. Alcohol oxidase is the first and rate-limiting enzyme in the methanol utilization pathway (MUT) [35]. It is located in the peroxidase body and is responsible for the oxidation of methanol to formaldehyde and hydrogen peroxide. Strain GL6 may not have this oxidase and lack the pathway of methanol metabolism. Therefore, it could not use methanol as the sole carbon source and had bad denitrification activity. For strain GL6, citrate was the most suitable carbon source to remove nitrate nitrogen, and the removal efficiency was 99.1% within 48 hours. The possible reason was that citrate could directly enter the tricarboxylic acid cycle and provide energy rapidly, which is a significant metabolic pathway of organic oxidation [36]. Because citrate was also inexpensive, citrate was employed in subsequent experiments.

### 3.4. Growth and Aerobic Denitrification Performance of Strain GL6

**Figure 4:**
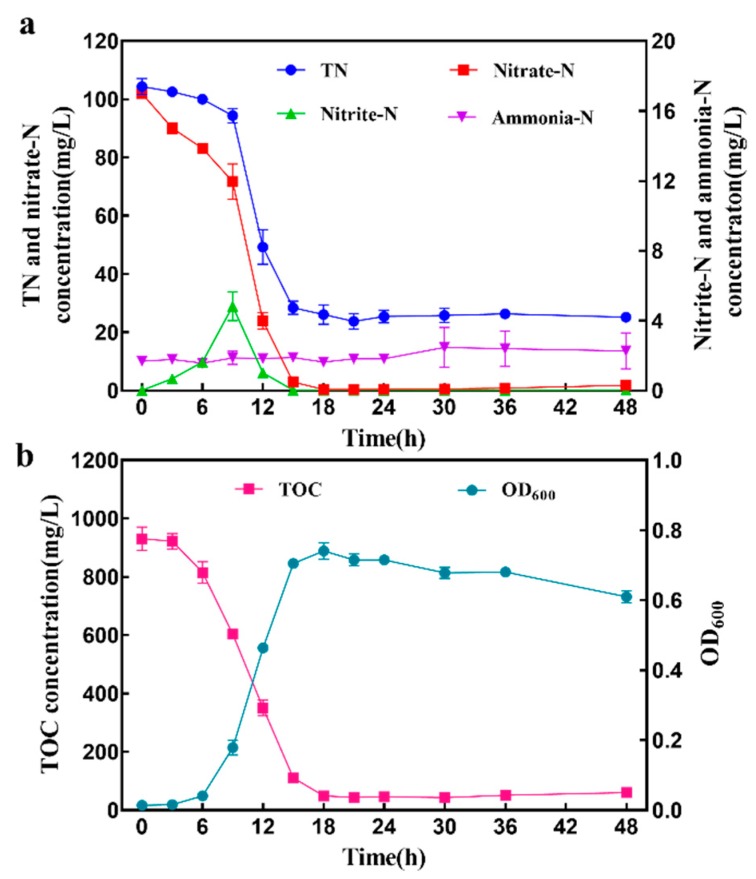
Changes in TN (total nitrogen), Nitrate-N, Nitrite-N, Ammonia-N (**a**) and TOC (total organic carbon), OD_600_ (**b**) by *Pseudomonas mendocina* strain GL6 in the AD medium. Values are given as mean ± SD for three replicates.

The ability of aerobic denitrification by strain GL6 was evaluated with potassium nitrate as the unique nitrogen source. Figure 4 describes the changes of the nitrogen compounds, OD_600_, and TOC with time. Herein, OD_600_ was employed as an indicator for the bacterial biomass, and TOC for the consumption of carbon sources. Within 6 h after inoculation, the OD_600_ barely increased and kept low values. This phenomenon indicated that bacteria grew slowly and acclimated to the liquid medium. This stage is the lag phase of strain GL6. From 6 to 18 h, the biomass (indicated by OD_600_) increased exponentially. Meanwhile, the nitrate-N concentration decreased significantly, as did the TN and TOC concentrations. After 18 h, the strain entered the stationary phase. OD_600_ reached the peak value of 0.741 and subsequently decreased slightly. TOC concentration decreased from 931.2 to 49.5 mg/L within 24 h with a removal efficiency of 94.7%, and TN removal efficiency was 74.4% at the same time. The results showed that the strain could remove nitrate and organic carbon simultaneously through heterotrophic aerobic denitrification.

From 18 to 24 h, the rate of decrease in nitrate-N concentration turned down. Nitrate-N concentration was basically unchanged after 24 h. The same trend in TN and TOC concentrations was observed. Strain GL6 removed 97% of nitrate-N within 15 h with an average removal rate of 6.61 mg (N)·L^−1^·h^−1^. The removal rate was higher than that of *Enterobacter cloacae* HNR with 4.58 mg (NO_3_^−^-N)·L^−1^·h^−1^ [12], *Klebsiella pneumoniae* CF-S9 with 2.20 mg (NO_3_^−^-N)·L^−1^·h^−1^ [29], and *Vibrio diabolicus* SF16 with 2.83 mg (NO_3_^−^-N)·L^−1^·h^−1^ [10]. Consequently, the excellent nitrate removal rate of strain GL6 was of value to wastewater treatment in practice. In addition, the nitrite-N concentration increased by degrees, and peaked at 4.83 mg/L after 9 h inoculation by strain GL6, then was almost completely removed at 15 h. The maximum nitrite accumulation of strain GL6 was lower than that of *Enterobacter* sp. strain FL with 31.0 mg/L [37] and *Pseudomonas stutzeri* strain XL-2 with 47.7 mg/L [38]. Ammonium stayed at a low concentration during the whole process. The ammonia-N concentration rose slightly in the decline phase, probably due to the release of dead cells [31].

Nitrogen balance was conducted with nitrate as the sole nitrogen source. As shown in Table 2, the nitrate-N concentration decreased from the initial 102.23 mg/L to the final 0.52 mg/L, occurring with the accumulation of trace amounts of NH_4_^+^-N, NO_2_^−^-N, and organic nitrogen. The comparison between the initial and final nitrogen concentrations can be calculated such that 20.2% of initial nitrate-N was converted into intracellular-N, and 5.37% was transformed into other nitrogen compounds (NH_4_^+^-N, NO_2_^−^-N, and organic nitrogen). Therefore, it is concluded that approximately 74.4% of the initial nitrogen was lost, probably converted into nitrogenous gases by aerobic denitrification. This result was consistent with the previous studies [9,31]. Therefore, the aerobic denitrifier has great potential for advanced treatment of wastewater treatment plant effluent.

### 3.5. Optimization of the Environmental Factors on the Aerobic Denitrification by Box–Behnken Design

It was found that aerobic nitrate removal of strain GL6 was greatly affected by four factors: C/N ratio, pH, shaking speed, and temperature. Moreover, the interactive effects of these factors were determined by RSM with a Box–Behnken design. The experimental tests and corresponding results are shown in Table S2. Based on the parameter estimation, the following quadratic polynomial equation was given to correlate the relationship between the four factors and TN removal rate.

Y = 77.89 + 9.7X_1_ + 17.59X_2_ + 9.89X_3_ − 8.26X_4_ + 10.42X_1_X_2_ + 5.99X_1_X_3_ + 9.42X_1_X_4_ + 2.29X_2_X_3_ − 7.53X_2_X_4_ + 8.28X_3_X_4_ − 26.41X_1_^2^ − 29.49X_2_^2^ − 24.19X_3_^2^ − 15.68X_4_^2^

where Y is TN removal rate and X_1_, X_2_, X_3_, and X_4_ are the coded values of C/N ratio, pH, shaking speed, and temperature, respectively.

The correlation coefficient (R^2^) of this regression model was 0.864 and the probability value (P) was 0.0007 (Table S3). The F-value of 6.35 indicated that the overall model was significant, as there was only a 0.07% chance that an F-value this large could occur due to noise. The result indicated that C/N ratio, pH, and shaking speed were significant factors, while temperature was an insignificant factor. The optimum conditions for strain GL6 were determined to be a C/N ratio of 11.2, a pH of 7.76, a shaking speed of 133 rpm, and a temperature of 27.8 °C by solving the regression equation.

**Figure 5:**
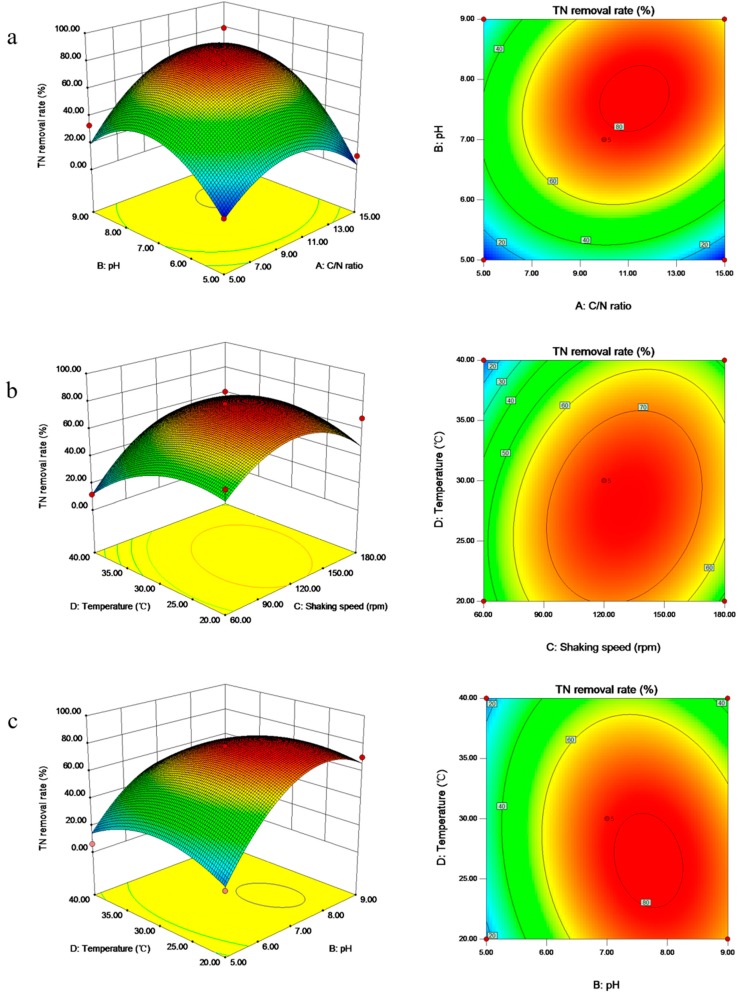
The response surface plots and corresponding contour plots of total nitrogen (TN) removal efficiency as a function of (**a**) C/N and pH; (**b**) shaking speed and temperature; (**c**) pH and temperature.

By means of three-dimensional response surface curves and contours, it was convenient to find out the optimum values of the variables for TN removal. Figure 5a displays the response surface and contours of the TN removal rate as a function of C/N ratio and pH as independent variables. The semispherical response surface of the TN removal rate gradually increased when the C/N ratio rose from 5.00 to 11.3, while the TN removal rate demonstrated a little reduction as the C/N ratio further raised. This phenomenon was in accordance with the previous research that denitrification capability decreased under extremely low or high C/N ratio [39]. Meanwhile, the TN removal rate enhanced as pH rose from 5.00 to 7.68, and then gradually decreased above a pH value of 7.68. In addition, the TN removal rate went down sharply at pH less than 6.00, suggesting that relatively acidic conditions may inhibit the aerobic denitrification. Thus, the optimum C/N ratio and pH were determined to be 11.26 and 7.68, respectively. Meanwhile, the optimal TN removal rate was achieved with a maximum of 82.12%.

Figure 5b depicts the effects of shaking speed and temperature on the TN removal rate. From the curve, it could be found that the pattern of response rose with shaking speed rising from 60 to 130.03 rpm and temperature rising from 20.0 to 27.8 °C, then it would be in a gradual downward tendency in some other region. Shaking speed reflected the DO (dissolved oxygen) concentration during the denitrification process. The relationship between shaking speed and the DO concentration of the solution is positively correlated. When the shaking speed was 130 rpm (DO: 5.81mg/L) and the temperature was 27.8 °C, the maximum value of TN removal rate was 79.6%. The optimum temperature range of aerobic denitrification was 25~37 °C, which was generally the optimum temperature for bacterial activity [40]. The optimum temperature of 27.8 °C was consistent with that reported in literature, such as *Pseudomonas stutzeri* strain ZF31 [31] and *Enterobacter* sp. CC76 [41].

Figure 5c shows the effects of two significant factors, pH and temperature, on the TN removal rate. The response surfaces demonstrate that the TN removal rate exceeded 70.00% at a pH range of 6.96–8.74 and a temperature range of 20.0–30.6 °C. The optimization values for these factors were found to be 7.68 for pH and 26.5 °C for temperature, with the TN removal rate of 82.3%. In general, the microbial growth was favored in the range of pH 6.00 to 9.00 [13]. The optimum pH value of 7.68 was in agreement with the previous report that the optimal pH for aerobic denitrification was neutral or alkalescent (7.00–8.00) [40]. It was noteworthy that the removal rate of TN could exceed 65.0% under the conditions of lower temperature (i.e., 20 °C) and pH value (6.63–9.00). This result suggested that strain GL6 might have potential to restore polluted water at low temperature.

### 3.6. Assessment of Strain GL6 for Application in Wastewater Treatment Plant Effluent

Bioaugmentation technology refers to the methods of improving wastewater treatment effect by adding microorganisms with specific functions in the biological treatment system, which promotes the removal of certain pollutants in polluted water [42]. The characteristics of nitrate nitrogen removal by heterotrophic aerobic denitrification bacteria in shaking flask medium culture conditions had been widely reported [12,31,37]. However, few studies concerned the removal of nitrate nitrogen by the bacteria in wastewater treatment plant effluent. In this study, the potential application of the isolate was investigated.

**Figure 6:**
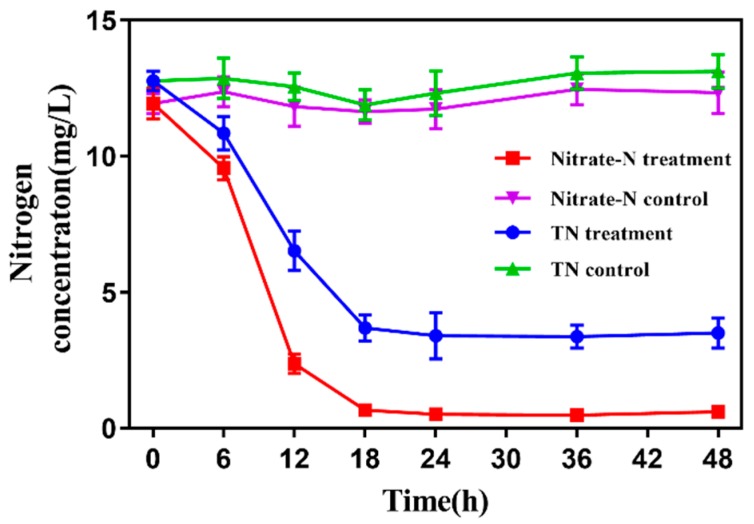
TN and nitrate in effluent of wastewater treatment plant inoculated with *P. mendocina* strain GL6 (treatment) compared with the control (without inoculation). Values represent the mean ± SD for three replicates.

*Pseudomonas mendocina* strain GL6 was inoculated into the wastewater with the optimum conditions above (i.e., a C/N ratio of 11.2, a pH of 7.76, a shaking speed of 133 rpm, and a temperature of 27.8 °C). As shown in Figure 6, the NO_3_^−^-N concentration decreased from 11.94 to 0.52 mg/L within 24 h with a removal efficiency of 95.6%, and TN removal efficiency was 73.6% at the same time. Meanwhile, NO_2_^−^-N could not be detected and NH_4_^+^-N concentration was insignificant in the whole experimental period. In the controls, there was almost no removal of NO_3_^−^-N and TN during the assay. Therefore, strain GL6 has potential application in the advanced treatment of wastewater treatment plant effluent. Nevertheless, subsequent experiments should be carried out to discuss the strategies of bioaugmentation and the survival capability of the strain GL6 in the practical wastewater treatment system [43].

## 4. Conclusions

*Pseudomonas mendocina* strain GL6 was newly isolated from the surface flow constructed wetland. The denitrification genes *napA, nirK, norB*, *and nosZ* were amplified. The optimum conditions for aerobic denitrification by strain GL6 were sodium citrate as the carbon source, C/N ratio of 11.2, pH of 7.76, shaking speed of 133 rpm, and temperature of 27.8 °C. Moreover, strain GL6 was inoculated into wastewater treatment plant effluent and showed the nitrate nitrogen removal rate of 95.6% and the TN removal rate of 73.6%. Thus, *P. mendocina* strain GL6 exhibited potential applications for the bioremediation of polluted water.

## Figures and Tables

**Table 1:** PCR primers used for denitrifying genes amplification.

Target Gene	Primer	Primer Sequence (5′→3′)	Product Size (bp)	Reference
*napA*	NAP1	TCTGGACCATGGGCTTCAACCA	876	[20]
	NAP2	ACGACGACCGGCCAGCGCAG		
*nirK*	nirK1F	GGMATGGTKCCSTGGCA	514	[21]
	nirK5R	GCCTCGATCAGRTTRTGGTT		
*nirS*	nirS cd3AF	GTSAACGTSAAGGARACSGG	425	[22]
	nirS R3cd	GASTTCGGRTGSGTCTTGA		
*norB*	cnorB2F	GACAAGNNNTACTGGTGGT	389	[23]
	cnorB6R	GAANCCCCANACNCCNGC		
*nosZ*	nosZ1527F	CGCTGTTCHTCGACAGYCA	250	[24]
	nosZ1773R	ATRTCGATCARCTGBTCGTT		

PCR: polymerase chain reaction; NAP: periplasmic nitrate reductase.

**Table 2:** Nitrogen balance of nitrate removal by strain GL6 in 24 h under aerobic conditions.

Substance	Initial TN (mg/L)	Final Nitrogen Concentration (mg/L)				Intracellular-N (%)	TN Removal (%)
		NH_4_^+^-N	NO_3_^−^-N	NO_2_^−^-N	Organic-N		
Nitrate	102.23 ± 0.21	1.82 ± 0.14	0.52 ± 0.29	0.02 ± 0.02	3.13 ± 0.65	20.63 ± 1.32	≈74

TN: total nitrogen.

## Supplementary Materials

The following are available online at http://www.mdpi.com/1660-4601/16/3/364/s1, Table S1: Level of variables and their values in Box–Behnken experimental design, Table S2: The Box–Behnken experimental design along with the corresponding responses, Table S3: The least-squares fit and the parameter estimates.
