# Supplementary material for: Characterization of Aerobic Denitrifying Bacterium Pseudomonas mendocina Strain GL6 and Its Potential Application in Wastewater Treatment Plant Effluent
Source: Int J Environ Res Public Health. 2019 Jan 28;16(3):364. doi: 10.3390/ijerph16030364 (PMC6388282; doi:10.3390/ijerph16030364)
Supplement: Supplementary file 1 [file ijerph-16-00364-s001.pdf]

Supplementary Materials

Characterization of an aerobic denitrifying bacterium *Pseudomonas mendocina* strain GL6 and its potential application in wastewater treatment plant effluent

Wen Zhang, Cheng Yan, Jianing Shen, Ruping Wei, Yan Gao, Aijun Miao, Lin Xiao, Liuyan Yang*

(State Key Laboratory of Pollution Control and Resource Reuse, School of the Environment, Nanjing University, Nanjing 210023, P. R. China)

***Corresponding author.** Tel. /fax: +86 25 8968 0257

E-mail address: yangly@nju.edu.cn

Table S1

Level of variables and their values in Box-Behnken experimental design

		Level of variables		
Independent variables	Unit	-1	0	1
C/N ratio	-	5	10	15
pH	-	5	7	9
Shaking speed	rpm	60	120	180
Temperature	ºC	20	30	40

Table S2

The Box-Behnken experimental design along with the corresponding responses

Runs	C/N ratio	Initial pH	Shaking speed	Temperature	TN removal rate (%)
1	10	7	120	30	78.07
2	10	7	120	30	78.21
3	15	7	120	40	47.2
4	10	7	60	40	11.61
5	10	5	120	20	12.95
6	5	7	120	40	4.71
7	10	9	120	20	70.35
8	10	7	120	30	77.71
9	10	9	180	30	26.79
10	5	7	180	30	27.23
11	10	7	120	30	77.56
12	15	7	180	30	48.5
13	5	7	60	30	14.08
14	15	5	120	30	10.19
15	10	9	120	40	33.43
16	15	7	120	20	31.81
17	5	7	120	20	27.01
18	10	5	180	30	7.3
19	10	7	180	40	60.47
20	10	5	60	30	9.97
21	10	9	60	30	20.3
22	10	7	180	20	68.12
23	10	7	120	30	77.92
24	15	7	60	30	11.38
25	5	5	120	30	5.77
26	10	7	60	20	52.39
27	15	9	120	30	79.29
28	10	5	120	40	6.15
29	5	9	120	30	33.21

Table S3

The least-squares fit and the parameter estimates

Factor	Coefficient Estimate	F value	*p* value Prob > F
Model	77.89	6.35	0.0007**
A-C/N ratio	9.70	5.39	0.0359*
B-pH	17.59	17.73	0.0009**
C-Shaking speed	9.89	5.61	0.0328*
D-Temperature	-8.26	3.91	0.0682
AB	10.42	2.07	0.1720
AC	5.99	0.69	0.4214
AD	9.42	1.70	0.2138
BC	2.29	0.10	0.7563
BD	-7.53	1.08	0.3156
CD	8.28	1.31	0.2715
A^2	-26.41	21.61	0.0004**
B^2	-29.49	26.95	0.0001**
C^2	-24.19	18.13	0.0008**
D^2	-15.68	7.62	0.0153*

**Very significant (p value < 0.01).

*Significant (0.01 < p value < 0.05).
